# Photorespiration differs among *Arabidopsis thaliana* ecotypes and is correlated with photosynthesis

**DOI:** 10.1093/jxb/ery274

**Published:** 2018-07-25

**Authors:** Nicholas J Tomeo, David M Rosenthal

**Affiliations:** Ohio University, Department of Environmental and Plant Biology, Athens, OH, USA

**Keywords:** *Arabidopsis thaliana*, heritability, *J*_max_, mesophyll conductance, natural selection, photorespiration, photosynthetic efficiency, *V*_Cmax_

## Abstract

A greater understanding of natural variation in photosynthesis will inform strategies for crop improvement by revealing overlooked opportunities. We use *Arabidopsis thaliana* ecotypes as a model system to assess (i) how photosynthesis and photorespiration covary and (ii) how mesophyll conductance influences water use efficiency (WUE). Phenotypic variation in photorespiratory CO_2_ efflux was correlated with assimilation rates and two metrics of photosynthetic capacity (i.e. *V*_Cmax_ and *J*_max_); however, genetic correlations were not detected between photosynthesis and photorespiration. We found standing genetic variation—as broad-sense heritability—for most photosynthetic traits, including photorespiration. Genetic correlation between photosynthetic electron transport and carboxylation capacities indicates that these traits are genetically constrained. Winter ecotypes had greater mesophyll conductance, maximum carboxylation capacity, maximum electron transport capacity, and leaf structural robustness when compared with spring ecotypes. Stomatal conductance varied little in winter ecotypes, leading to a positive correlation between integrated WUE and mesophyll conductance. Thus, variation in mesophyll conductance can modulate WUE among *A. thaliana* ecotypes without a significant loss in assimilation. Genetic correlations between traits supplying energy and carbon to the Calvin–Benson cycle are consistent with biochemical models, suggesting that selection on either of these traits would improve all of them. Similarly, the lack of a genetic correlation between photosynthesis and photorespiration suggests that the positive scaling of these two traits can be broken.

## Teaser

Photosynthesis and photorespiration covary, but this relationship is not fully understood. Natural variation among *Arabidopsis thaliana* ecotypes offers an ideal system for interrogating the physiological co-ordination of photosynthesis and photorespiration.

## Introduction

Despite a near complete description of the photorespiratory pathway, availability and characterization of knockout mutants for many of the enzymes in the pathway (Maurino and Peterhansel, 2010), and thorough understanding of the response of photorespiration to environmental stimuli, relatively little is known about natural genetic variation in photorespiration (Nunes-Nesi *et al.*, 2016). Photorespiration reduces the photosynthetic conversion efficiency by almost half (Zhu *et al.*, 2010), providing motivation to modify it to improve crop yields (Long *et al.*, 2015; Ort *et al.*, 2015; Walker *et al.*, 2016). Photorespiration occurs when 2-phosphoglycolate is formed by the oxygenation of ribulose-1,5-bisphosphate (RuBP) by Rubisco. Briefly, two 2-phosphoglycolate molecules are metabolized via the C_2_ photorespiratory pathway, resulting in the return of one phosphoglycerate to the Calvin–Benson cycle, but also the release of one CO_2_ molecule, and consumption of both ATP and reducing equivalents (Maurino and Peterhansel, 2010; Peterhansel *et al.*, 2010). Additionally, photorespiration is sensitive to environmental change as the ratio of oxygenation to carboxylation reactions by Rubisco increases with temperature (Jordan and Ogren, 1984; Bernacchi *et al.*, 2001), and decreases with increasing [CO_2_].

From an ecological and evolutionary perspective, natural genetic variation for traits among habitats and populations may reveal some of the history of differential selection pressures on those populations (Antonovics and Bradshaw, 1968; Geber and Dawson, 1990; Dudley, 1996; Franks *et al.*, 2007; Flood *et al.*, 2016). Natural genetic variation has informed our unraveling of the mechanistic and genetic basis of several ecologically and agronomically important plant traits, but is underused when it comes to enhancing photosynthetic efficiency to improve crop yields (Flood *et al.*, 2011; Driever *et al.*, 2014; Nunes-Nesi *et al.*, 2016; Orr *et al.*, 2016). Natural genetic variation has provided many yield-enhancing traits including pathogen and pest resistance (Foster *et al.*, 1991; Hill *et al.*, 2006), improved heat tolerance (Maestri *et al.*, 2002), yield enhancement through increasing harvest indices (Hay, 1995), and the proliferation of dwarfing genes in cereals (Hedden, 2003), to name a few.

A better understanding of how natural selection has altered net carbon assimilation (*A*_N_) (Flood *et al.*, 2011) and photorespiration in divergent habitats and niches provides a framework for enhancing *A*_N_ in crop species because natural populations illustrate the extent of phenotypic space occupied by successful trait combinations (Donovan *et al.*, 2011). Genetic variation exists for a wide array of photosynthetic traits in wild (Geber and Dawson, 1997; Brouillette *et al.*, 2014; Momayyezi and Guy, 2017) and some crop plants (Barbour *et al.*, 2010; Giuliani *et al.*, 2013; Gu *et al.*, 2014; Tomeo and Rosenthal, 2017). In *Arabidopsis thaliana*, *A*_N_ and associated traits differ substantially among wild populations (Brosché *et al.*, 2010; Easlon *et al.*, 2014; van Rooijen *et al.*, 2015). Ecotypes exhibit variability in their stomatal responsiveness to [CO_2_] (Takahashi *et al.*, 2015) and other stimuli (Aliniaeifard and Van Meeteren, 2014) which alter *A*_N_. There is standing heritable genetic variation among ecotypes for light use efficiency (i.e. quantum yield of PSII) (van Rooijen *et al.*, 2015), integrated water use efficiency (WUE; i.e. carbon isotope composition of leaves: δ^13^C)(McKay *et al.*, 2003; Easlon *et al.*, 2014), and both stomatal conductance and transpiration efficiency (Easlon *et al.*, 2014). However, the nature and magnitude of variation in photorespiration and covariation with photosynthesis are not fully resolved in crops and hardly explored in wild plants.

The biochemical model of photosynthesis (Farquhar *et al.*, 1980) provides a robust and well-tested system to study photosynthetic variation. Empirical fits to the model demonstrate that photosynthesis is commonly limited by both light and carbon supply under natural (Wullschleger, 1993) and agronomic conditions (Bernacchi *et al.*, 2005; Chen *et al.*, 2005). This co-limitation by carboxylation and RuBP regeneration, referred to as the ‘teeter–totter’ model, is effectively maintained by partitioning resources between carboxylation capacity (*V*_Cmax_) and electron transport capacity (*J*_max_) (Farquhar *et al.*, 2001). A consistent ratio is preserved between *V*_Cmax_ and *J*_max_ across differing environments (Walker *et al.*, 2014) even when redistribution of nitrogen between the traits would enhance photosynthesis (Evans, 1989; Zhu *et al.*, 2007; Kromdijk and Long, 2016). At least one study has indicated that *V*_Cmax_ and *J*_max_ are genetically correlated in wild plants (Geber and Dawson, 1997). If a genetic correlation between *V*_Cmax_ and *J*_max_ is a universal phenomenon, this may constrain independent selection on the traits—away from the observed, consistent ratio—to maximize photosynthesis.

In this study, we set out to demonstrate the extent of natural phenotypic variation and covariation for leaf-level C_3_ photosynthetic physiology and photorespiration and to identify the existence and nature of genetic correlations among these traits. *Arabidopsis thaliana* ecotypes originating from diverse geographic and climatic regions (Table 1) were selected to represent a range of variation in rosette-level gas exchange parameters (Easlon *et al.*, 2014). Evidence suggests that photosynthesis and photorespiration are co-regulated (Bauwe *et al.*, 2012; Timm *et al.*, 2016), leading to the hypothesis that they should be positively correlated, though even the magnitude of intraspecific variation for photorespiration within and among natural populations is not fully understood. Here we hypothesize that: (i) carbon and energy supplies to the Calvin–Benson cycle are co-ordinated at both genetic and phenotypic levels; and (ii) variation in photorespiration exists and is positively correlated with carbon and energy supplies to the Calvin–Benson cycle. Easlon *et al.* (2014) suggested that stomatal conductance was of greater consequence to WUE than *A*_N_ in *A*. *thaliana* ecotypes, but mesophyll conductance was also implicated as driving some of the variation in WUE. Therefore, we test the hypothesis (iii) that WUE—either intrinsic (i.e. the ratio of *A*_N_ to stomatal conductance of water vapor: *A*_N_/*g*_sH_) or integrated (δ^13^C)—is correlated negatively with stomatal conductance, but positively with mesophyll conductance and net assimilation.

**Table 1.** A list of all of the ecotypes used in this study

Name	Accession	Latitude	Longitude	MAT	MAP	Habit
Ag-0	CS76430	45.0000	1.3000	11.8	887	Winter
Bil-5	CS76709	63.3240	18.4840	2.9	615	Winter
Bur-0	CS76734	54.1000	–6.2000	9.3	953	Spring
Eden-1	CS76826	62.8770	18.1770	3.3	655	Winter
Kas-1	CS903	35.0000	77.0000	–9.1^*a*^	74^*a*^	Winter
Knox-18	CS76530	41.2816	–86.6210	9.7	975	Spring
Ler-1	CS77021	47.9840	10.8719	8.0	962	Spring
NFA-10	CS77126	51.4108	–0.6383	9.8	701	Spring
Omo2-3	CS77149	56.1400	15.7800	7.9	546	Winter
Sq-8	CS76604	51.4083	–0.6383	9.8	701	Spring
Tamm-2	CS76610	60.0000	23.5000	5.1	611	Winter
Ts-1	CS76615	41.7194	2.9306	16.1	644	Spring
Tsu-1	CS77390	34.4300	136.3100	14.9	2385	Spring
Ws-2	CS76631	52.3000	30.0000	6.7	624	Spring

The names, accession numbers, and latitude/longitude of origin are referenced to the Arabidopsis Biological Resource Center. Mean annual temperature (MAT) and mean annual precipitation (MAP) were taken from the bioclim database (www.worldclim.org, last accessed 7 August 2018; Hijmans *et al.*, 2005) using the given latitude/longitude locations with help from the rgdal (Bivand *et al.*, 2016) and raster (Hijmans, 2016) R packages. Habit refers to the life history strategy of ecotypes: winter habits require vernalization for flowering

^***a***^Available latitude and longitude are coarse for this ecotype. Due to the rugged terrain surrounding this location and the high variability in the local climate over small spatial scales, these climate values represent rough approximations.

## Materials and methods

### Plant material

Seeds of 14 *A. thaliana* ecotypes (Table 1), chosen to reflect as much photosynthetic trait variance as possible based on previous measurements (Easlon *et al.*, 2014), were obtained from the Arabidopsis Biological Resource Center (abrc.osu.edu; last accessed 7 August 2018). One plant of each ecotype was grown for one generation to collect seed in a common environment, minimizing maternal environmental effects. Seeds were sown in 160 ml pots in a moist 4:1 mixture of Metro-Mix 360 topsoil (Sun Gro Horticulture, Agawam, MA, USA) and fritted clay (Turface, Profile Products LLC, Buffalo Grove, IL, USA). Pots were placed in trays with ~1 cm of water, covered, and incubated in the dark at 4 °C for 7 d. Trays were transferred to a controlled-environment chamber set to a photosynthetic photon flux density (PPFD) at plant height of ~400 µmol photons m^–2^ s^–1^, day length of 12 h, and day:night temperature of 21:19 °C. At the six-leaf stage, pots were thinned to one plant, pots were rotated within trays weekly, and trays were rotated within the chamber every 3–4 d. Water was replaced as needed and supplemented with 0.5× Hoagland’s solution weekly. At 21 d post-germination, winter-annual ecotypes were transferred to a second growth chamber set to identical conditions, except that the temperature was set to constant 4 °C, where they were kept for 28 d before returning them to the original chamber. As a precautionary measure, 40 cm floral sleeves were placed around the pots before flowering. Once the majority of siliques on a plant reached maturity, the plant was placed in a separate tray without water, allowed to dry for several days, and seeds were harvested.

To facilitate leaf-level gas exchange measurements on rosette leaves, 160 ml pots were overfilled, with the soil mixture used above, and covered with a fiberglass screen to hold it in place. Plants were grown in six replicate blocks with each block contained on a single tray. Two trays (i.e. blocks) were grown simultaneously in a growth chamber set to a PPFD at plant height of ~450 µmol photons m^–2^ s^–1^ during 12 h days, day:night temperature of 21:18.5 °C, and relative humidity maintained between 60% and 80%. The additional four blocks were grown iteratively. Initially four seeds were sown in each pot and at the six- to eight-leaf stage were thinned to two plants arranged at opposite sides of the pot. Once newly developed leaves reached maximum width for a given ecotype, but always before bolting, one plant from each pot was measured.

### Gas exchange and associated measurements

The response of photosynthesis to intercellular [CO_2_] was measured at ambient and 1% O_2_. The youngest fully expanded leaf was placed in the 6400-40 fluorescence chamber (LI-6400XT Photosynthesis System, LI-COR Environmental, Lincoln, NE, USA) making sure not to damage the leaf, while ensuring contact with the thermocouple and covering as much of the 2 cm^2^ chamber as possible. Leaves were acclimated for ≥25 min to a PPFD of 850 µmol photons m^–2^ s^–1^ with 10% blue light, a vapor pressure deficit below 1.2 kPa, ambient [CO_2_] of 400 µmol CO_2_ mol^–1^ air, flow rate of 300 µmol air min^–1^, and block temperature controlled to hold leaf temperature at 25 °C. Preliminary light response curves indicated that all ecotypes were light saturated at 650 µmol photons m^–2^ s^–1^. Upon reaching steady-state conditions, a data point was logged and the [CO_2_] was changed iteratively along the sequence 400, 325, 250, 175, 100, 50, 400, 400, 500, 650, 950, 1250, and 1600 µmol CO_2_ mol^–1^ air. After reaching stable conditions at each CO_2_ set-point, gas exchange parameters and steady-state fluorescence (*F*_s_) were logged. Before proceeding to the next [CO_2_], a multiphase flash chlorophyll fluorescence routine was executed to determine the maximum (*F*_m_ʹ) fluorescence following recommended procedures (Loriaux *et al.*, 2013). Once the initial CO_2_–response curve at ambient [O_2_] was complete, the leaf was allowed to re-acclimate to ambient conditions until *A*_N_ and stomatal conductance returned to their initial steady-state values. Air from an N_2_ tank with 1% (v/v) O_2_ was piped through a humidifying system and connected to the air inlet on the 6400 console. A second CO_2_–response curve was performed with only subambient CO_2_ concentrations (400, 325, 250, 175, 100, and 50 µmol mol^–1^). At each CO_2_ set-point, *F*_s_ and *F*_m_ʹ were again estimated with the multiphase flash routine. As some leaves did not fill the gas exchange cuvette, a digital image of the leaf section in the cuvette and a ruler were captured and used to calculate the area with ImageJ (Schneider *et al.*, 2012), and all gas exchange parameters were recalculated.

Following gas exchange, leaf absorptance (α) was determined with a spectroradiometer connected to dual integrating spheres built into a leaf clamp (Jaz Spectroclip, Ocean Optics Inc., Dundee, FL, USA). Reflectance and transmittance were measured at three locations on the leaf used for gas exchange. The calculation of α was constrained to ±5 nm surrounding the light-emitting diode (LED) peaks of the 6400-40 light source (i.e. 470 nm and 665 nm), and was weighted to account for the light used during gas exchange, being composed of 10% blue and 90% red. Absorptance was calculated as 1–(reflectance+transmittance).

Leaf thickness (*T*_L_) was measured with digital calipers on the leaf of the rosette directly opposite that used for gas exchange. Then this leaf and 4–5 additional fully expanded leaves were sampled for determination of leaf mass per area (LMA) and leaf dry matter content (LDMC). The total area of these leaves was determined using ImageJ. The leaves were then massed for fresh weight. After drying at 65 °C for >72 h, the leaves were massed again for dry weight. LDMC was calculated as the ratio of dry to fresh mass. LMA was calculated as the ratio of dry mass to total leaf area. Dry leaves were then ground to a fine powder and analyzed for carbon, nitrogen, and ^13^C content at the University of Illinois. The stable carbon isotope ratio (^13^C to ^12^C) of leaf tissue relative to a standard is reported as δ^13^C in units of parts per thousand (‰).

### Analysis of gas exchange

Leaks in gas exchange systems can result in systematic biases due to the contrasting diffusional gradients at opposing ends of a CO_2_–response curve (Flexas *et al.*, 2007*a*; Rodeghiero *et al.*, 2007; Gong *et al.*, 2015). As a precaution against these biases, we measured apparent photosynthesis throughout identical CO_2_–response curves on heat-inactivated (*n*=13) leaves with petioles kept in water to maintain hydration. Apparent photosynthesis of these heat-inactivated leaves was then subtracted from all measured CO_2_–response curves, followed by updating the estimates of intercellular [CO_2_] (*C*_i_) given the new net assimilation rates (Flexas *et al.*, 2007*a*; Li-Cor Biosystems, 2012). While heat-inactivated leaves do not perfectly match the characteristics of living leaves, they should mimic the characteristics of leaves better than wet filter paper (Flexas *et al.*, 2007*a*), and many of the diffusional leaks in the 6400 system are probably upstream of the leaf chamber and therefore will be equally accounted for despite the material used to mimic intact leaves.

CO_2_–response curves were initially fit to the C_3_ biochemical model of photosynthesis (Farquhar *et al.*, 1980) to estimate mitochondrial respiration in the light (*R*_d_). Each curve was fit using the using the R package plantecophys (Duursma, 2015) and the estimate for *R*_d_ was extracted. Mean ecotype *R*_d_ values (see Supplementary Table 1 at *JXB* online) were calculated and used in the calculations below.

Electron transport rates through PSII measured combined with gas exchange and chlorophyll fluorescence supports alternative electron sinks, photosynthesis, and the photorespiratory pathway. If corrections for alternative electron sinks are implemented, we can partition the remaining electrons to their respective destinations in photosynthesis and photorespiration. We first quantified the quantum yields of CO_2_ fixation (Φ_CO2_) and PSII (Φ_PSII_) (Valentini *et al.*, 1995; Long and Bernacchi, 2003) as:

$$\Phi_{\text{CO2}}=\left(A_{\text{N}}+R_{\text{d}}\right)/(\text{aPPFD})$$ (1)

ΦPSII=(Fm'–Fs)/Fm' (2)

where *A*_N_ is the leak-corrected net assimilation rate, α is the leaf absorptance measured as above; PPFD, *F*_m_ʹ, and *F*_s_ were taken from the 6400 output. The relationship of Φ_CO2_ and Φ_PSII_ under non-photorespiratory conditions (1% O_2_) is linear (Genty *et al.*, 1989) with the intercept representing the share of electrons destined to alternative sinks and the slope indicating the number of electrons required for reducing a CO_2_ molecule *in vivo*. Assuming that this electron partitioning holds under photorespiratory conditions, Φ_PSII_ was calibrated (Φ_e_; Valentini *et al.*, 1995) as:

$$\text{At 1}\%{\text{ O}}_{\text{2}}:\Phi_{\text{PSII}}=k\Phi_{\text{CO2}}+b$$ (3)

$$\text{At 21}\%{\text{ O}}_{\text{2}}:\Phi_{\text{e}}=\text{4}\left(\Phi_{\text{PSII}}–b\right)/k$$ (4)

where *k* and *b* are the slope and intercept of the linear regression, respectively, and four is the theoretical number of electrons required for a single carboxylation (Long and Bernacchi, 2003). Φ_e_ in Equation 4 represents the quantum efficiency of PSII corrected for any alternative electron sinks. With Φ_e_ we calculated the total electron flux through PSII used to support both photosynthesis and photorespiration (*J*_T_):

$$J_{\text{T}}=\Phi_{\text{e}}\text{PPFD}$$ (5)

and

$$J_{\text{T}}=J_{\text{C}}+J_{\text{O}}$$ (6)

where *J*_C_ and *J*_O_ are the electron flows to carboxylation and oxygenation, respectively. Then assuming that four electrons are required for each CO_2_ carboxylation, and eight electrons for each CO_2_ release following an oxygenation event:

$$J_{\text{C}}=\text{4}\left(A_{\text{N}}+R_{\text{d}}+\text{PR}\right)$$ (7)

$$J_{\text{O}}=\text{8PR}$$ (8)

where PR is the photorespiratory CO_2_ release rate, calculated as:

$$\text{PR}=\left[J_{\text{T}}–\text{4}\left(A_{\text{N}}+R_{\text{d}}\right)\right]/\text{12}$$ (9)

We then insert Equation 9 into Equations 7 and 8 to partition electrons between the two pathways:

$$J_{\text{C}}=\left[J_{\text{T}}+\text{8}\left(A_{\text{N}}+R_{\text{d}}\right)\right]/\text{3}$$ (10)

$$J_{\text{O}}=\text{2}\left[J_{\text{T}}–\text{4}\left(A_{\text{N}}+R_{\text{d}}\right)\right]/\text{3}$$ (11)

Photorespiration can be estimated with other methods, each with assumptions, benefits, and drawbacks (Busch, 2013). We opted for the above method, which combines two independent data sources, gas exchange and fluorescence. Comparison with another model yielded comparable results (Supplementary Fig. S1).

A reliable estimate of *J*_T_ also allows for the calculation of mesophyll conductance (*g*_m_) with the variable-*J* method (Harley *et al.*, 1992):

$$g_{\text{m}}=A_{\text{N}}/<C_{\text{i}}–\{\text{G}*\left[J_{\text{T}}+\text{8}\left(A_{\text{N}}+R_{\text{d}}\right)\right]/\left[J_{\text{T}}–\text{4}\left(A_{\text{N}}+R_{\text{d}}\right)\right]\}>$$ (12)

where Γ* is the photosynthetic CO_2_ compensation point in the absence of *R*_d_. There are several values of Γ* in the literature for *A*. *thaliana*, ranging from 3.3 Pa to 5.4 Pa (Kebeish *et al.*, 2007; Flexas *et al.*, 2007*b*; Cousins *et al.*, 2011; Walker and Cousins, 2013; Walker *et al.*, 2013; Weise *et al.*, 2015). Calculation of mesophyll conductance is sensitive to variation in Γ* (Pons *et al.*, 2009) so we estimated *g*_m_ with Γ* values of 3.64 Pa (Walker *et al.*, 2013) and 4.47 Pa (Weise *et al.*, 2015) since these fall within, but span, most of the range in estimates. The absolute value of *g*_m_ estimates was greater with Γ* of 4.47 Pa. The correlation of *g*_m_ estimates with Γ* of 3.64 Pa and 4.47 Pa was high (*r*=0.989, data not shown) and no differences in the resulting genetic variance of *g*_m_ were detected. Therefore we chose to use the value of Γ* from Walker *et al.* (2013) since it falls in between the extremes of Γ* for *A. thaliana* and is closest to the commonly used value from tobacco (*Nicotiana tabacum*) (Bernacchi *et al.*, 2002). Theoretically, with the variable-*J* method, *g*_m_ can be estimated at any *C*_i_ where net assimilation (*A*_N_) is carboxylation limited. However, since *g*_m_ is often observed to vary with *C*_i_, we estimated *g*_m_ from a common, near-ambient [CO_2_] of 325 µmol mol^–1^ (*C*_i_ range 240–285). Carboxylation should be limiting at this concentration, it should more accurately reflect the *C*_i_ of leaves with an intact boundary layer, and we saw no significant difference in estimates at ambient [CO_2_]s of 325 µmol mol^–1^ or 400 µmol mol^–1^ (data not shown). With a known *g*_m_, the [CO_2_] in the chloroplast (*C*_c_) was calculated as:

$$C_{\text{c}}=C_{\text{i}}–A_{\text{N}}/g_{\text{m}}$$ (13)

With values of *C*_c_ we again fit the biochemical model of C_3_ photosynthesis (Farquhar *et al.*, 1980) using plantecophys (Duursma, 2015) to estimate the maximum carboxylation capacity (*V*_Cmax_) and maximum electron transport capacity (*J*_max_). Since there is no way to ensure the PPFD was truly saturating for all *A*_N_–*C*_i_ curves, *J*_max_ is reported as *J*_850_ (i.e. subscripted with the measurement PPFD of 850 µmol photons m^–2^ s^–1^) (Buckley and Diaz-Espejo, 2015).

### Statistical analysis

All computations and statistical analyses were performed with the R statistical computing environment (R Core Team, 2015). Models with ecotype and replication block as random effects were fit with the lmer function (Bates *et al.*, 2015) for each trait using restricted maximum likelihood. The significance (*P*<0.05) of ecotype was determined with likelihood ratio tests between models with and without the ecotype effect. Given the number of traits investigated, we corrected all *P*-values to account for multiple testing using the false discovery rate (Benjamini and Hochberg, 1995).

For traits with a significant ecotype effect, genetic variation for each was then determined by partitioning variance to ecotype (*V*_G_), replication block (*V*_E_), and residual variation (*V*_R_). Broad-sense heritability (*H*^2^) was calculated as *H*^2^=*V*_G_/(*V*_G_+*V*_E_/6+*V*_R_), where *V*_E_ was divided by six to account for the six replicate blocks in the experimental design. Additionally, for each trait with a significant ecotype effect, we calculated best linear unbiased predictors (BLUPs) as the model-predicted ecotype values. Phenotypic correlations between traits were calculated as Pearson product–moment correlations with all observations of the two traits. Likewise, genetic correlations were calculated as the Pearson product–moment correlation of trait BLUPs. Again, *P*-values were adjusted to account for the large number of comparisons (Benjamini and Hochberg, 1995). Trait values in ecotypes with spring (*n*=8) versus winter (*n*=6) growth habits were compared with Welch’s unequal variance *t*-tests to account for the unequal sample sizes.

## Results

We observed substantial phenotypic variation for leaf-level physiological traits in the 14 *A. thaliana* ecotypes assessed here. Ecotypes are naturally occurring genetically distinct populations allowing separation of phenotypic variation into its genetic, environmental, and residual components and calculation of broad-sense heritability of traits (Table 3). The ecotypes differed for all traits examined; that is, genetics played a significant role in determining the phenotypic range of a given ecotype. Stomatal conductance to CO_2_ (*g*_sc_; see Table 2 for all trait abbreviations and their units) and mesophyll conductance (*g*_m_) differed 2.4- and 2.5-fold, respectively, across all observations (ranging 0.0724–0.259 µmol CO_2_ m^–2^ s^–1^ and 0.356–1.20 µmol CO_2_ m^–2^ s^-1^ Pa^–1^), while variation among ecotype BLUPs (i.e. genotypic variation) was 57% and 41%, respectively. Conductance through the full CO_2_ diffusion path (*g*_tot_) varied 1.8-fold phenotypically (range: 0.0248–0.0707 µmol CO_2_ m^–2^ s^-1^) and 0.4-fold genotypically. Reductant supply to the photosynthetic reactions varied similarly, though at lower magnitudes. Total, calibrated, linear electron transport (*J*_T_) rates varied 1.4-fold phenotypically (range: 62.7–150 µmol *e*^–^ m^–2^ s^–1^) and 21% genotypically. Total electron transport was partitioned to electrons destined for carboxylation (*J*_C_) and oxygenation (*J*_O_) reactions, which varied 1.4- and 0.80-fold phenotypically (ranging 42.2–99.8 µmol *e*^–^ m^–2^ s^–1^ and 19.0–57.5 µmol *e*^–^ m^–2^ s^–1^, respectively), or 19% and 29% genotypically, respectively. It follows that *A*_N_ differed 1.6-fold phenotypically (range: 6.97–18.3 µmol CO_2_ m^–2^ s^–1^) and 21% genotypically. Photorespiration (PR) also varied, >2-fold phenotypically (range: 2.38–7.19 µmol CO_2_ m^–2^ s^–1^) and 29% genotypically. As implied by the presence of genotypic variation, estimates of broad-sense heritability were significant for all traits above (Table 3).

**Table 2.** List of all trait abbreviations used and their units

Trait	Abbreviation	Units
Ambient CO_2_ concentration	*C*_a_	µmol CO_2_ mol^–1^ air
Intercellular CO_2_ concentration	*C*_i_	µmol CO_2_ mol^–1^ air
Chloroplast CO_2_ concentration	*C*_c_	µmol CO_2_ mol^–1^ air
Stomatal conductance to water vapor	*g*_sH_	mol H_2_O m^–2^ s^–1^
Stomatal conductance to CO_2_	*g*_sc_	mol CO_2_ m^–2^ s^–1^
Mesophyll conductance	*g*_m_	µmol CO_2_ m^–2^ s^–1^ Pa^–1^
Total CO_2_ conductance	*g*_tot_	mol CO_2_ m^–2^ s^–1^
Total linear electron transport	*J*_T_	µmol *e*^–^ m^–2^ s^–1^
Electron transport to carboxylation	*J*_*C*_	µmol *e*^–^ m^–2^ s^–1^
Electron transport to oxygenation	*J*_O_	µmol *e*^–^ m^–2^ s^–1^
Maximum carboxylation capacity	*V*_Cmax_	µmol CO_2_ m^–2^ s^–1^
Maximum electron transport capacity	*J*_max_ (*J*_850_^*a*^)	µmol *e*^–^ m^–2^ s^–1^
Net assimilation rate	*A*_N_	µmol CO_2_ m^–2^ s^–1^
Photorespiratory CO_2_ efflux rate	PR	µmol CO_2_ m^-2^ s^-1^
Intrinsic water use efficiency	*A*_N_/*g*_sH_	µmol CO_2_ mol^–1^ H_2_O
Integrated water use efficiency	*δ* ^13^C	‰
Leaf nitrogen content	*N*_mass_	%
Nitrogen per unit leaf area	*N*_area_	g m^–2^
Leaf dry mass per area	LMA	g m^–2^
Leaf dry matter content	LDMC	g dry g^–1^ wet
Leaf thickness	*T*_L_	mm

^*a*^The symbol *J*_850_ is used throughout to indicate that *J*_max_ was estimated at a photon flux density of 850 µmol m^–2^ s^–1^.

**Table 3.** Variance components and heritability estimates for the primary traits investigated

Trait	*V*_G_	*V*_E_	*V*_R_	*H* ^2^	*P*
*g*_sc_	3.72e-4	7.19e-5	8.44e-4	0.30	*
*g*_m_	0.00733	0.00243	0.0147	0.33	***
*g*_tot_	2.35e-5	6.55e-6	5.71e-5	0.29	**
*J*_T_	75.8	57.4	218	0.25	**
*J*_C_	25.8	20.3	88.3	0.22	*
*J*_O_^*a*^	16.2	9.27	37.5	0.29	**
*V*_Cmax_	21.3	16.7	60.6	0.25	**
*J*_850_	148	55.9	228	0.38	***
*A*_N_	0.915	0.552	3.22	0.22	*
PR^*a*^	0.253	0.145	0.586	0.29	**
*A*_N_/*g*_s_	52.7	15.0	55.2	0.48	***
δ^13^C	0.930	0.0144	0.291	0.76	***
*N*_mass_	0.123	0.00357	0.0504	0.71	***
*N*_area_	0.0219	0.00110	0.0116	0.65	***
LMA	5.72	0.450	2.88	0.67	***

The genotypic variance (*V*_G_), environmental variance (*V*_E_), residual variance (*V*_R_), broad-sense heritability (*H*^2^), and significance (*P*) are presented for each of the traits. All variance components were estimated with restricted maximum likelihood. *V*_E_ represents the portion of phenotypic variance attributable to replication blocks, and *V*_R_ is the remaining residual variance in the models. Significance values are from likelihood ratio tests comparing models with and without the random effect of ecotype, where *P<0.05, **P<0.01, and ***P<0.001 after correcting the false discovery rate for multiple testing.

^*a*^Note that PR and *J*_O_ are direct numeric transformations of one another.

Leaf structural and WUE traits also varied among ecotypes. Nearly half of the variation in both LMA and LDMC was contributed by genetics, with 46% and 39% of variation among ecotype BLUPs for the two traits that varied ~1- and 0.75-fold phenotypically. Intrinsic WUE (*A*_N_/*g*_sH_) differed 1.5-fold across all observations and 42% (i.e. 21 µmol CO_2_ mol^–1^ H_2_O) among ecotypes. Leaf carbon isotope composition (δ^13^C), a metric of integrated WUE, differed 4.8‰ among observations and 2.9‰ among BLUPs. Broad-sense heritability estimates were generally higher for WUE and structural traits than for photosynthetic traits (Table 3).

**Fig. 1.**
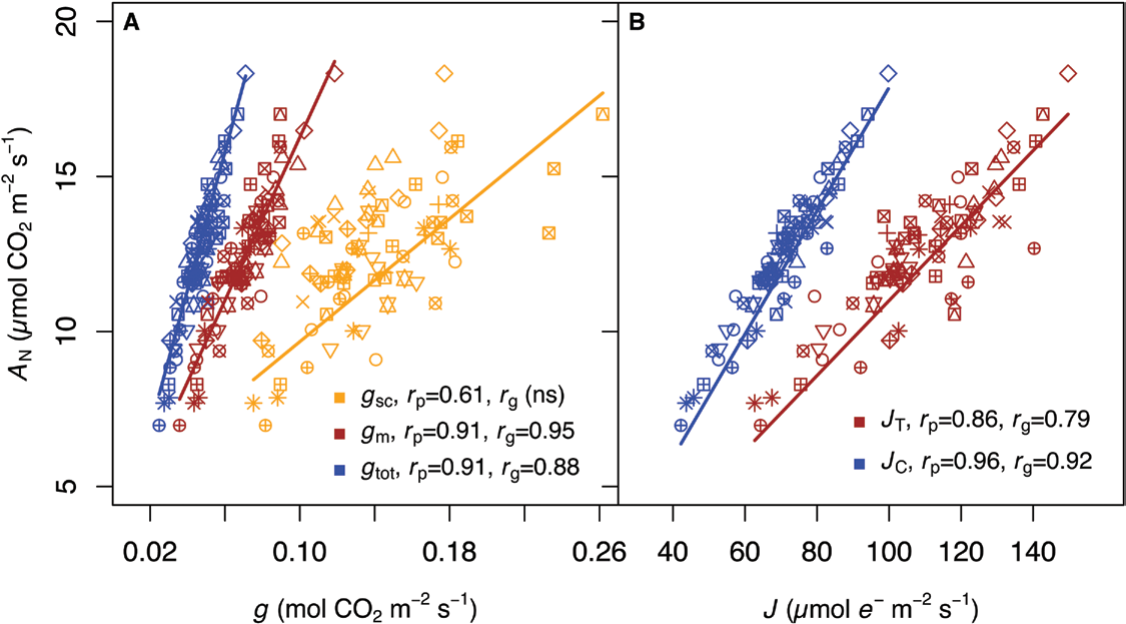
Relationship between net CO_2_ assimilation (*A*_N_) and (A) conductance of CO_2_ and (B) electron transport. Conductance of CO_2_ is presented as stomatal (*g*_sc_; right most fit line), mesophyll (*g*_m_; middle fit line), and total conductance from the boundary layer to chloroplasts (*g*_tot_; left fit line). Electron transport is presented as total calibrated-linear (*J*_T_; right fit line), and as the partition of electrons utilized as reductant in carboxylation reactions (*J*_C_; left fit line). Phenotypic (*r*_p_) and genetic (*r*_g_) correlations, where significant (*P*<0.01 for all here), are presented; (ns) is not significant. Each ecotype is represented by a unique symbol. Lines are mixed-model fits with ecotype identity and replication block treated as random effects.

**Fig. 2.**
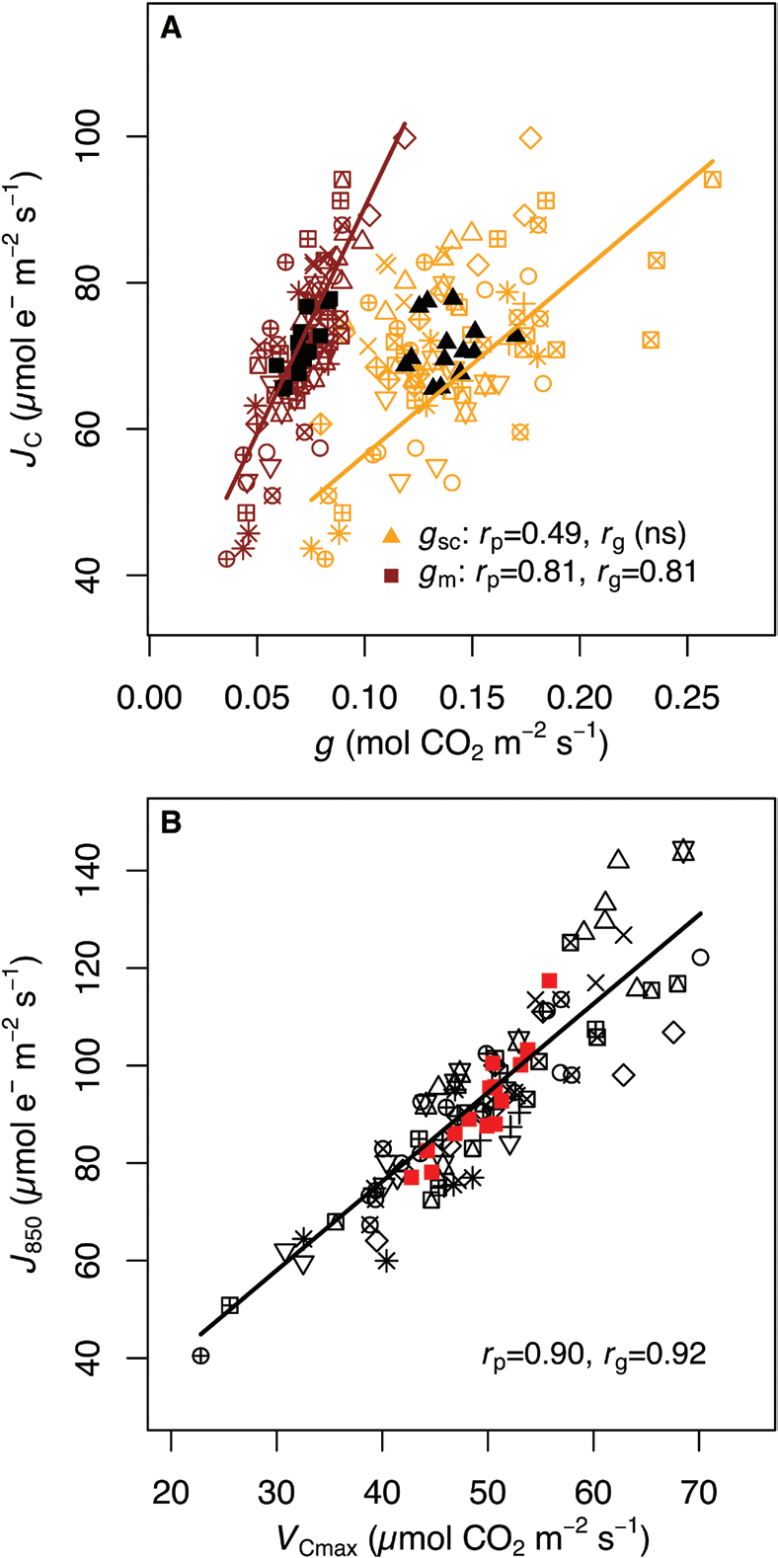
The relationship between (A) electron transport driving carboxylation (*J*_C_) with stomatal (*g*_sc_; right fit line) and mesophyll (*g*_m_; left fit line) conductance to CO_2_, and (B) maximum electron transport (*J*_850_) and carboxylation (*V*_Cmax_) capacities. Each ecotype is designated by a unique open symbol. Filled symbols (solid triangles and squares in A; solid squares in B) represent ecotype best linear unbiased predictors. Lines are mixed-model fits to the bivariate relationships with ecotype identity and replication as random effects. When significant, phenotypic (*r*_p_) and genetic (*r*_g_) correlations are presented (*P*<0.01 for all); (ns) is not significant.

Physiological leaf traits were by and large correlated with one another both phenotypically (*r*_p_) and genotypically (*r*_g_) (Table 4). Phenotypic correlations signify that the traits are co-ordinated in some way, while genetic correlations signify that some portion of that co-ordination results from genetic differentiation among populations (i.e.ecotypes). Phenotypically the traits supplying the Calvin–Benson cycle with CO_2_, *g*_sc_ and *g*_m_, were correlated (*r*_p_=0.49, *P*<0.001), though the genetic correlation was not significant (Table 4; *P*>0.1). All CO_2_ conductance traits (*g*_sc_, *g*_m_, and *g*_tot_) were correlated with *A*_N_, with the sole exception of the genetic correlation between *A*_N_ and *g*_sc_ (Fig. 1). Phenotypic and genetic correlations between *J*_T_ and *A*_N_ were both significant (*r*_p_=0.86, *r*_g_=0.79, *P*<0.001 for both), but were lower than the correlation with *A*_N_ after partitioning electrons specifically to carboxylation, namely *J*_C_ (*r*_p_=0.96, *r*_g_=0.92, *P*<0.001 for both). Both *g*_sc_ and *g*_m_ were phenotypically correlated with *J*_T_ (*g*_sc_: *r*_p_=0.37, *P*<0.01; *g*_m_: *r*_p_=0.67, *P*<0.001) and *J*_C_ (*g*_sc_: *r*_p_=0.49, *P*<0.001; *g*_m_: *r*_p_=0.81, *P*<0.001) (Fig. 2 A), but only *g*_m_ was genetically correlated with electron transport (*J*_T_: *r*_g_=0.66, *P*<0.05; *J*_C_: *r*_g_=0.81, *P*<0.01). Maximum biochemical capacities for carboxylation (*V*_Cmax_) and electron transport (*J*_850_) were tightly correlated both phenotypically (*r*_p_=0.90, *P*<0.001) and genetically (*r*_g_=0.92, *P*<0.001) (Fig. 2B).

**Table 4.** Pearson product–moment correlation matrix of physiological traits

	*g*_sc_	*g*_m_	*g*_tot_	*J*_T_	*J*_C_	*J*_O_	*V*_Cmax_	*J*_850_	*A*_N_	PR	*A*_N_/*g*_s_	δ^13^C
*g*_sc_	–	**0.49**	**0.79**	**0.37**	**0.49**	0.17	**0.43**	**0.25**	**0.61**	0.17	**–0.69**	**-0.36**
*g*_m_	0.43	–	**0.92**	**0.67**	**0.81**	**0.40**	**0.76**	**0.71**	**0.91**	**0.40**	0.15	**0.28**
*g*_tot_	**0.77**	**0.91**	–	**0.63**	**0.78**	**0.35**	**0.73**	**0.60**	**0.91**	**0.35**	–0.21	0.02
*J*_T_	–0.11	**0.66**	0.41	–	**0.97**	**0.94**	**0.72**	**0.64**	**0.86**	**0.94**	**0.25**	**0.40**
*J*_C_	0.10	**0.81**	**0.62**	**0.97**	–	**0.83**	**0.79**	**0.71**	**0.96**	**0.83**	0.18	**0.35**
*J*_O_	–0.39	0.38	0.08	**0.94**	**0.82**	–	**0.53**	**0.49**	**0.64**	**1.0**	**0.32**	**0.44**
*V*_Cmax_	0.29	**0.82**	**0.70**	**0.65**	**0.77**	0.42	–	**0.90**	**0.82**	**0.53**	0.19	**0.26**
*J*_850_	0.07	**0.68**	0.50	0.57	**0.64**	0.42	**0.92**	–	**0.71**	**0.49**	**0.33**	**0.34**
*A*_N_	0.44	**0.95**	**0.88**	**0.79**	**0.92**	0.53	**0.81**	**0.63**	–	**0.64**	0.07	**0.23**
PR	–0.39	0.38	0.08	**0.94**	**0.82**	**1.0**	0.42	0.42	0.53	–	**0.32**	**0.44**
*A*_N_/*g*_s_	**–0.71**	0.30	–0.10	**0.74**	**0.62**	**0.82**	0.40	0.48	0.34	**0.82**	–	**0.67**
δ^13^C	**–0.71**	0.31	–0.10	**0.69**	0.58	**0.76**	0.38	0.49	0.31	**0.76**	**0.97**	–

The phenotypic correlations are presented above the diagonal, and genotypic correlations are below. Genetic correlations were estimated from ecotype best linear unbiased predictors. Correlations in bold were statistically significant (*P*<0.05) after correcting for multiple testing. Abbreviations are as in Table 2. Note that since PR and *J*_O_ are direct numeric transformations of one another, they are perfectly correlated

**Fig. 3.**
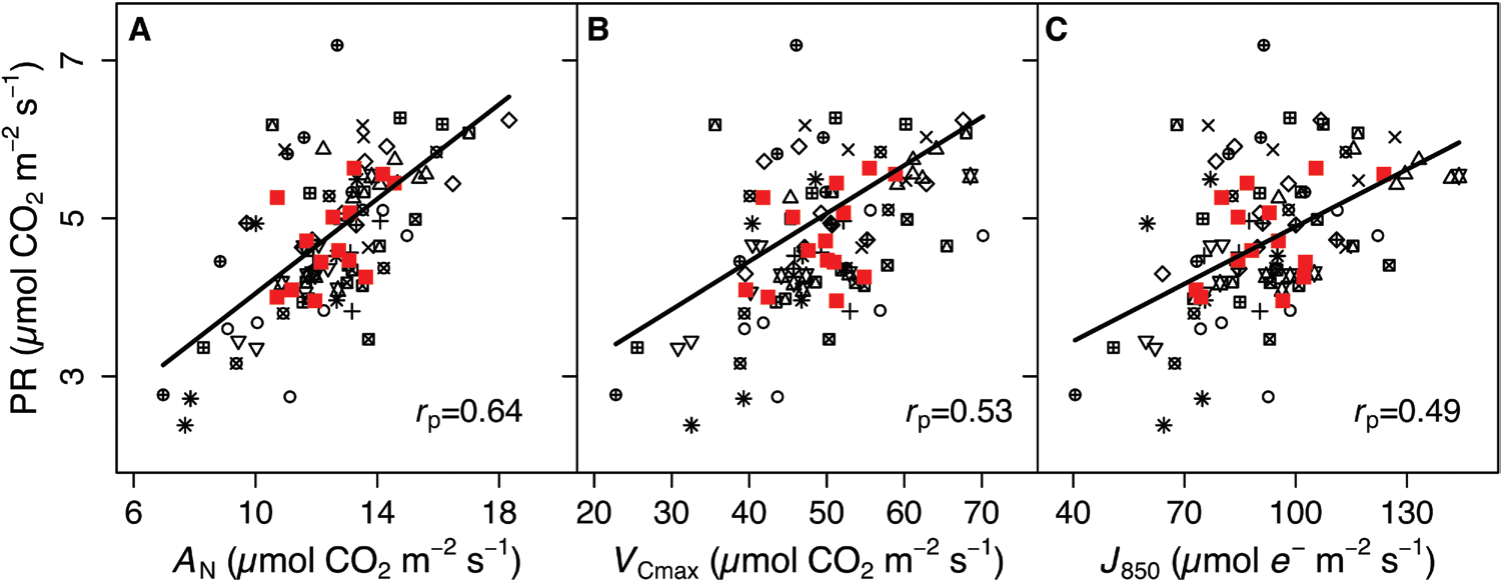
Photorespiratory CO_2_ efflux (PR) as a function of (A) net assimilation (*A*_N_), (B) the maximum carboxylation capacity (*V*_Cmax_), and (C) maximum electron transport capacity (*J*_850_). Phenotypic correlations (*r*_p_) are presented (*P*<0.001 for all); no genetic correlations were significant after correcting for multiple testing. Ecotypes are represented by unique symbols and ecotype means are overplotted as solid squares. Fit lines are drawn from mixed-models with ecotype and replication block as random effects.

To estimate photorespiration (PR), we partitioned the portion of *J*_T_ used to support PR (*J*_O_), and then calculated PR from *J*_O_. While *J*_O_ and PR represent different aspects of physiology, they are calculated from the same combination of traits (i.e. *A*_N_, *R*_d_, and *J*_T_), only differing with respect to the stoichiometric coefficients used in their calculation such that *J*_O_ and PR are directly proportional to one another. Therefore, comparisons with both traits yield equivalent statistical results—albeit with differing parameter estimates. We present results for PR only since we are interested specifically in carbon losses associated with photorespiratory CO_2_ efflux. We detected a significant correlation between *A*_N_ and PR phenotypically (*r*_p_=0.64, *P*<0.001; Fig. 3A), though the genetic correlation was not significant after controlling for multiple testing (*r*_g_=0.53, 0.1>*P*>0.05). Since *A*_N_ is an input parameter in the calculation of PR, some correlation of *A*_N_ and PR is expected. We further test the physiological correlation of photosynthesis with photorespiration by comparing the biochemical parameters that underlie photosynthesis, namely *V*_Cmax_ and *J*_850_, with PR. The phenotypic correlations of PR with *V*_Cmax_ (*r*_p_=0.53, *P*<0.001) and *J*_850_ (*r*_p_=0.49, *P*<0.001) were lower than with *A*_N_ directly, but a clear positive relationship remained (Fig. 3B, C). No genetic correlations were detected between PR and *V*_Cmax_ or *J*_850_ (Table 4).

**Fig. 4.**
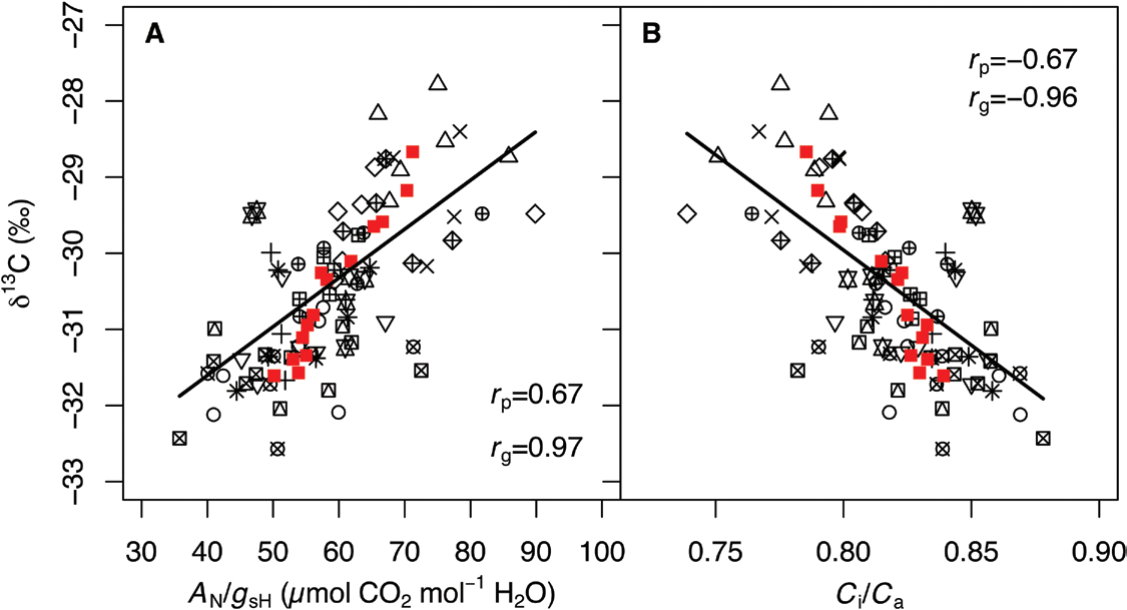
Leaf-level integrated water use efficiency (δ^13^C) by (A) intrinsic water use efficiency (*A*_N_/*g*_sH_) and (B) the ratio of intercellular to atmospheric [CO_2_] (*C*_i_/*C*_a_). Phenotypic and genetic correlations were significant (*P*<0.001) for both. Each ecotype is represented by a unique symbol, and solid squares are ecotype best linear unbiased predictors. Fit lines represent mixed-model fits with replication and ecotype as random effects.

**Fig. 5.**
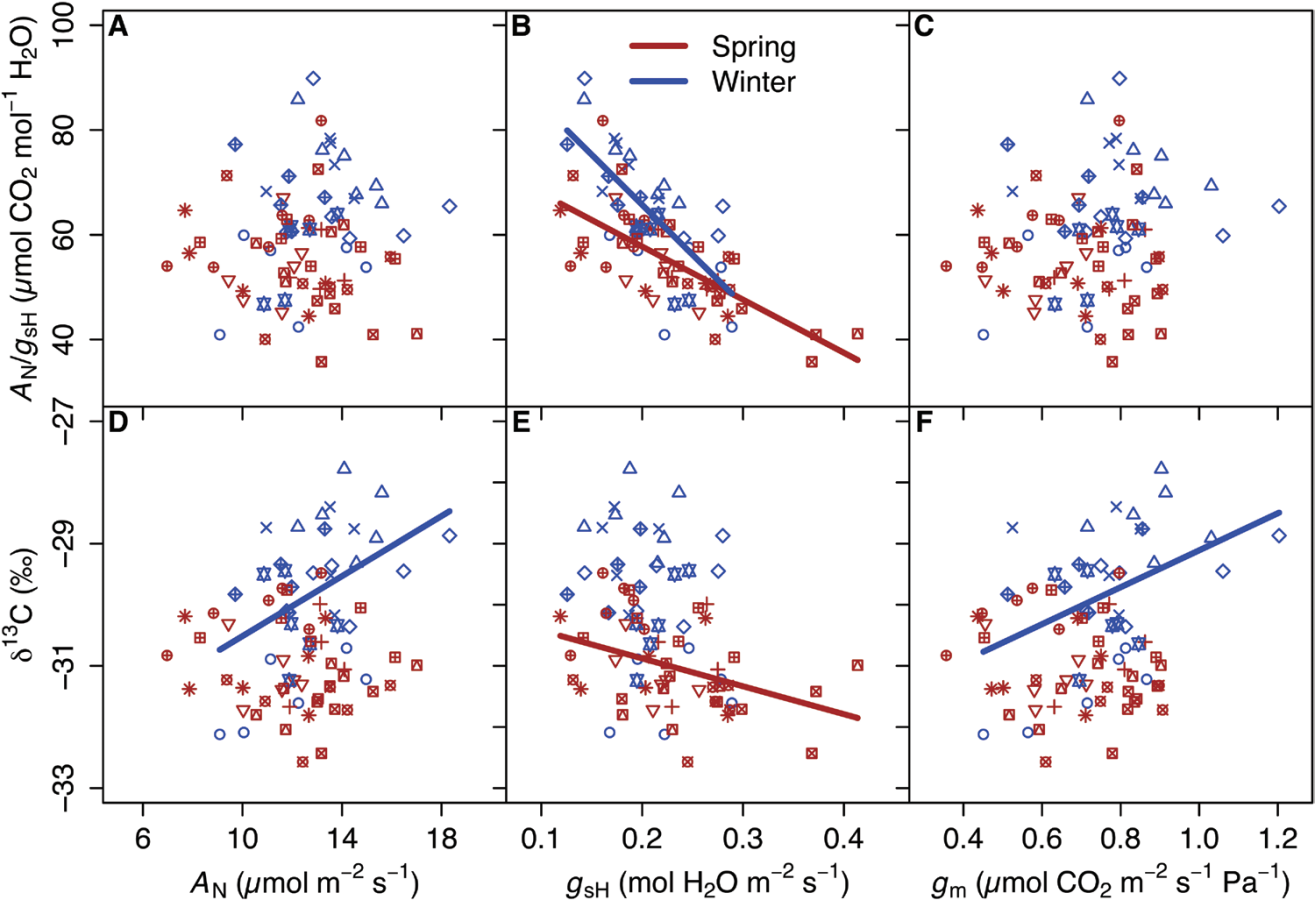
Relationships between water use efficiency metrics and components effecting water use efficiency. Correlations between intrinsic water use efficiency (*A*_N_/*g*_sH_) and (A) *A*_N_, (B) stomatal conductance to water vapor (*g*_sH_), and (C) mesophyll conductance (*g*_m_). Integrated water use efficiency (δ^13^C) is likewise correlated with (D) *A*_N_, (E) *g*_sH_, and (F) *g*_m_. Ecotypes are represented by unique symbols and are shaded according to their life history strategy. Fit lines, presented when significant (*P*<0.05), are linear regressions for spring (B, E) or winter (B, D, F) subsets of the data.

Intrinsic WUE (*A*_N_/*g*_sH_) is determined by the interaction of *A*_N_ and *g*_sH_, and therefore by any trait influencing *A*_N_ or *g*_sH_ (e.g. *g*_m_ and *V*_Cmax_). Therefore, we assessed the extent to which each of these traits contributes individually to *A*_N_/*g*_sH_ and/or δ^13^C. Both metrics of WUE were phenotypically correlated (*r*_p_=0.67, *P*<0.001) and demonstrated an even stronger genetic correlation with each other (*r*_g_=0.971, *P*<0.001)(Fig. 4A). The phenotypic (*r*_p_= –0.67, *P*<0.001) and genetic (*r*_g_= –0.96, *P*<0.001) correlations of δ^13^C with the ratio of intercellular to ambient [CO_2_] (*C*_i_/*C*_a_) mirrored those with *A*_N_/*g*_sH_ (Fig. 4B). Net assimilation rates were not correlated with *A*_N_/*g*_sH_, and only a weak phenotypic correlation was found with δ^13^C (*r*_p_=0.276, *P*<0.05) (Table 4; Fig. 5A, D). Evidence of *g*_sH_ influencing WUE was stronger: phenotypic and genetic correlations were observed between *g*_sH_ and *A*_N_/*g*_sH_ (*r*_p_= –0.69, *P*<0.001; and *r*_g_= –0.71, *P*<0.05) and δ^13^C (*r*_p_= –0.36, *P*<0.01; and *r*_g_= –0.71, *P*<0.05) (Table 4; Fig. 5B, E). The influence of *g*_m_ mirrored that of *A*_N_ in that it was only weakly correlated phenotypically with δ^13^C (*r*_p_=0.276, *P*<0.05) (Table 4; Fig. 5C, G). The full path of CO_2_ diffusional conductance, *g*_tot_, was not associated with either metric of WUE (Table 4). Ecotype growth habit (spring or winter annual type) altered relationships with WUE. The correlation of δ^13^C with *A*_N_, or *g*_m_, was only significant for winter annual types (Fig. 5D, F), and with *g*_sH_ only for spring types (Fig. 5E).

**Fig. 6.**
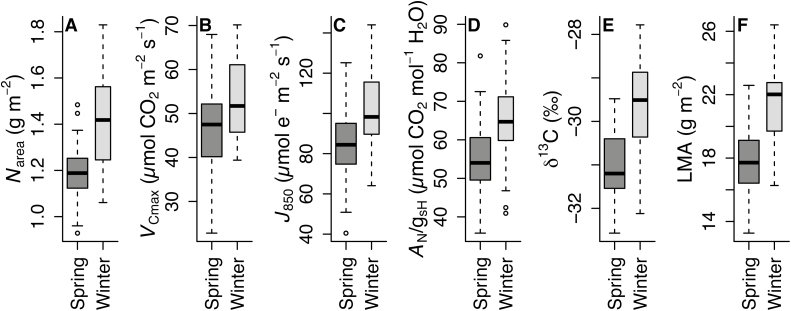
Comparisons of trait values in ecotypes exhibiting spring or winter annual life history strategies. The traits are (A) nitrogen content per unit leaf area (*N*_area_), (B) maximum carboxylation capacity (*V*_Cmax_), (C) maximum electron transport capacity (*J*_850_), (D) intrinsic water use efficiency (*A*_N_/*g*_sH_), (E) integrated water use efficiency (δ^13^C), and (F) leaf mass per area (LMA). Winter annuals (*n*=6) have significantly (*P*<0.01) greater trait values than spring annuals (*n*=8) following unequal variance *t*-tests. Eight of the 14 ecotypes exhibit the spring habit. Box width is scaled to the relative sample size in each group (*n*=46 for spring, *n*=34 for winter).

The ecotypes used in this study originate from areas spanning a large latitudinal range, and thus areas differing widely in variables that covary with latitude (e.g. temperature and precipitation; Table 1). Ecotypes were also split between those exhibiting winter and spring annual life history strategies, and, with the exception of Kas-1, winter annual types tend toward higher latitudes (Table 1). Winter annual types had higher *g*_m_ (*t*=2.7, *P*<0.05; not shown), *N*_area_ (*t*=6.1, *P*<0.001), *V*_Cmax_ (*t*=3.1, *P*<0.01), *J*_850_ (*t*=3.1, *P*<0.001), LMA (*t*=7.7, *P*<0.001), WUE (*A*_N_/*g*_sH_: *t*=4.3, *P*<0.001; δ^13^C: *t*=5.7, *P*<0.001) (Fig. 6), *T*_L_ (*t*=4.3, *P*<0.001), and LDMC (*t*=7.1, *P*<0.001) (Supplementary Fig. S1).

## Discussion

We found a strong co-ordination between assimilation and photorespiration as we show that photorespiration was phenotypically correlated with net carbon assimilation (*A*_N_), and with the maximum capacities for carboxylation and electron transport (i.e. *V*_Cmax_ and *J*_850_; Fig. 3). Molecular crosstalk between the Calvin–Benson cycle and the photorespiratory pathway indicates that the two processes should manifest at the physiological scale as co-ordination between photorespiratory CO_2_ efflux (PR) and *A*_N_. Indeed, the Calvin–Benson cycle and photorespiratory pathway are interlinked to such an extent at the molecular level that they are referred to holistically as a ‘supercycle’ (Timm *et al.*, 2016). If flux through the photorespiratory pathway is too slow, the concentration of 2-phosphoglycolate will rise until it inhibits regeneration of RuBP through the Calvin–Benson cycle, ultimately suppressing carbon fixation (Igamberdiev *et al.*, 2004; Bauwe *et al.*, 2012). In addition, the photorespiratory intermediates glyoxylate (Chastain and Ogren, 1989) and glycerate (Schimkat *et al.*, 1990) act as signals to the Calvin–Benson cycle, allowing rapid co-regulation of the two pathways (Timm *et al.*, 2016). Recent work now suggests that the scaling of *A*_N_ and PR is also maintained as a result of photorespiratory nitrate assimilation providing a secondary carbon sink in the form of amino acid production (Busch *et al.*, 2018).

Genetic variation for photosynthetic traits is commonly observed in wild species (Comstock and Ehleringer, 1992; Geber and Dawson, 1997; Arntz and Delph, 2001; Geber and Griffen, 2003; Brouillette *et al.*, 2014), crops (Reynolds *et al.*, 2000; Gu *et al.*, 2012; Liu *et al.*, 2012; Tomeo and Rosenthal, 2017), and specifically in *A. thaliana* (McKay *et al.*, 2003; Aliniaeifard and Van Meeteren, 2014; Easlon *et al.*, 2014; Takahashi *et al.*, 2015; van Rooijen *et al.*, 2015). Natural variation in photorespiration has been reported in a few crops including tobacco (Zelitch and Day, 1973), alfalfa (Peterschmidt, 1980), and wheat (Aliyev, 2012). We demonstrate that heritable genetic variation for photorespiration also exists for *A. thaliana* (Table 3). Aliyev (2012) found diurnal and seasonal co-ordination between *A*_N_ and PR when contrasting high- and low-yielding wheat (*Triticum aestivum*) varieties, and that greater *A*_N_ and PR were associated with higher yielding varieties. It seems likely that genetic variation for PR exists in most crops. If and how natural variation in photorespiration exists in wild plants and whether this is associated with fitness is an interesting and an open question. If the significant broad-sense heritability for PR exists across plants, the implication is that natural selection could alter this trait if there are measurable fitness effects of PR. We could not detect a genetic correlation between PR and *A*_N_ (or *V*_Cmax_, or *J*_850_) here, but additional analyses are needed to assess the nature and extent of linkage between *A*_N_ and PR more broadly and if or how selection could act on either trait independently.

### Co-ordination of photosynthetic drivers

Across the *A. thaliana* ecotypes, there was broad co-ordination among photosynthetic traits. We expected to see phenotypic correlations among traits supporting photosynthesis because *V*_Cmax_ and *J*_max_ are co-ordinated at various scales reflecting optimization of nitrogen distribution among photosynthetic proteins to balance photosynthetic limitation between carboxylation and RuBP regeneration (Wullschleger, 1993; Geber and Dawson, 1997; Walker *et al.*, 2014). Co-ordination should also be expected between traits influencing CO_2_ diffusion from the intercellular air spaces to Rubisco (i.e. *g*_sc_, *g*_m_, and *g*_tot_), the rates of electron transport through the light-dependent reactions of photosynthesis (i.e. *J*_T_ and *J*_C_), and carboxylation capacity (i.e. *V*_*Cmax*_). Indeed, *g*_sc_, *g*_m_, *J*_T_, *J*_C_, *J*_850_, and *V*_Cmax_ were all phenotypically intercorrelated (Table 3). Both *g*_m_ versus *J*_C_ and *V*_Cmax_ versus *J*_850_ also exhibited strong genetic correlations. This indicates that the essential co-ordination of the photosynthetic light-dependent and light-independent reactions supporting the co-limitation of photosynthesis by RuBP carboxylation and regeneration is not simply a product of optimal allocation (Evans, 1989; Hikosaka and Terashima, 1995), but rather is constrained by genetics, or alternatively has been constrained by selection (Donovan *et al.*, 2011). Genetic correlations result from pleiotropy or linkage disequilibrium and, while we cannot disentangle the contribution of each of these mechanisms here, this experimental system provides a path for doing so in future studies. Regardless of the mechanism, the strength of the genetic correlation between *V*_Cmax_ and *J*_850_ here, and in at least one other study (Geber and Dawson, 1997), indicates that selection on either trait should simultaneously alter the other.

### Drivers of variation in water use efficiency (WUE)

Variation in leaf-level WUE emerges from the complex interaction of a suite of traits. We expected to see *g*_m_ positively correlated with intrinsic WUE (*A*_N_/*g*_sH_) due to the positive relationship between *A*_N_ and *g*_m_, and because *g*_m_ should increases *A*_N_ without increasing *g*_sH_. A strong case has been made for greater *g*_m_ driving greater intrinsic WUE (Flexas *et al.*, 2013), though this idea depends on *A*_N_ being a strong driver of variation in *A*_N_/*g*_sH_. A previous study in *A. thaliana* (Easlon *et al.*, 2014) and one in soybean (*Glycine max*) (Tomeo and Rosenthal, 2017) indicate that in herbaceous annual species with relatively high *g*_sH_, *A*_N_/*g*_sH_ is determined to a greater extent by variation in *g*_sH_ than *A*_N_—at least under conditions where water is not limiting.

Several complications arise when interpreting *A*_N_/*g*_sH_ as WUE because it is a ratio of traits that covary; these complications are partially avoided by also considering integrated WUE (i.e. δ^13^C, the stable carbon isotope ratio of leaf tissue relative to a standard source). With leaves grown in the same environment, leaf δ^13^C is indicative of discrimination against ^13^C, which relates to the ratio of intercellular to ambient [CO_2_] (*C*_i_/*C*_a_) over the lifetime of carbon accumulation in the leaf (Farquhar *et al.*, 1982; Dawson *et al.*, 2002; Seibt *et al.*, 2008). Any change in either *A*_N_ or *g*_sH_ will alter *C*_i_ independently of *C*_a_, so the *C*_i_/*C*_a_ ratio is related to *A*_N_/*g*_sH_, leading to the interpretation of δ^13^C as proportional to the mean leaf lifetime WUE. Rubisco discrimination against ^13^C is strictly speaking responsive to the [CO_2_] in the chloroplast (*C*_c_), and therefore is also dependent on *g*_m_ (Seibt *et al.*, 2008). All else being equal, higher *g*_m_ leads to greater *C*_c_ and ^13^C discrimination (and more negative δ^13^C). Variation for δ^13^C in *A. thaliana* is well documented in the literature among ecotypes (Nienhuis *et al.*, 1994; McKay *et al.*, 2003; Easlon *et al.*, 2014) and inbred lines (Nienhuis *et al.*, 1994; Juenger *et al.*, 2005). As in other studies (Schuster *et al.*, 1992; Easlon *et al.*, 2014), we show that broad-sense heritability for δ^13^C was high among the ecotypes (Table 3) and δ^13^C was correlated closely with both *A*_N_/*g*_sH_ and *C*_i_/*C*_a_ (Fig. 4).

### Variation in trait co-ordination: winter versus spring ecotypes

Considering all ecotypes, regardless of growth habit, we confirm earlier results (Easlon *et al.*, 2014) that δ^13^C is more closely correlated with *g*_sH_ than with *A*_N_. However, when we consider each growth habit separately, this alters the interpretation of which traits drive variation in δ^13^C. Specifically, among spring annual ecotypes, only *g*_sH_ was significantly related to δ^13^C. In contrast, for those with winter annual habits, *g*_sH_ was not correlated with δ^13^C, but *g*_m_ and *A*_N_ were positively correlated with δ^13^C (Fig. 5). Thus, higher *g*_m_, and not lower *g*_sH_, led to higher integrated WUE in winter annuals. The latter result is interesting as it provides some evidence that the influence of *g*_m_ on *A*_N_ can increase WUE (e.g. Flexas *et al.*, 2013, 2016).

WUE is well described as a trade-off with respect to the time required to transition from vegetative to reproductive growth in annual weedy plants including *A. thaliana* (McKay *et al.*, 2003) and *Polygonum arenastrum* (Geber, 1990; Geber and Dawson, 1990). In both species there is a continuum of traits conferring a drought avoidance to tolerance trade-off: fast developing genotypes have lower WUE and reach the reproductive transition quickly to avoid drought, whereas slower growing genotypes have greater WUE allowing them to tolerate drought more effectively (Geber, 1990; Geber and Dawson, 1990; McKay *et al.*, 2003). In our study, ecotypes with the winter habit exhibited a greater phenotypic and genotypic range of WUE (as *A*_N_/*g*_sH_ or δ^13^C; Figs 5, 6), therefore sampling the phenotypic space of the drought avoidance versus tolerance axis more completely than spring habit ecotypes. The added variance in the WUE traits was modulated by roughly equal ranges of *A*_N_ and *g*_m_ for winter and spring ecotypes, but winter ecotypes presented approximately half the range in *g*_sH_. Several studies have suggested that selection to improve WUE by enhancing *g*_m_ is contingent upon maintaining *g*_sH_ relatively constant (Flexas *et al.*, 2013, 2016), with a report on soybean cultivars indicating that a strong correlation between *g*_sH_ and *g*_m_ impedes our ability to alter WUE through selection on *g*_m_ (Tomeo and Rosenthal, 2017). However, here we show that natural selection may have already followed this proposed route (i.e. modulating *g*_m_ to alter WUE) in an array of genotypes with a range of WUEs.

Winter habit ecotypes possess a suite of traits that differ from spring ecotypes, including greater structural robustness (i.e. *T*_L_, LMA, and LDMC), instantaneous and integrated WUE *A*_N_/*g*_sH_ and δ^13^C, leaf N (*N*_area_), mesophyll conductance (*g*_m_), and photosynthetic capacity (*V*_Cmax_ and *J*_850_). We do not know if winter ecotypes have higher Rubisco content or activation, but one could predict that selection would favor this in plants adapted to shorter days. We hypothesize that thicker leaves (i.e. greater *T*_L_) in *A. thaliana* arise due to a thicker palisade mesophyll layer which allows for a larger area of mesophyll cell surface exposed to intercellular airspace. Greater LMA is consistent with a greater number of mesophyll cells per unit leaf area (Milla-Moreno *et al.*, 2016) enabling higher *N*_area_, *V*_Cmax_, and *J*_850_. Taken together, this suggests that a greater number of parallel diffusion paths exist for CO_2_ into the mesophyll cells (e.g. Milla-Moreno *et al.*, 2016) of winter ecotypes, explaining their greater *g*_m_. Likewise, plants with greater *g*_m_ have a higher [CO_2_] in their chloroplasts, slightly altering the ratio of CO_2_ to O_2_, potentially driving some of the variation in photorespiration and *V*_Cmax_ detected here.

**Fig. 7.**
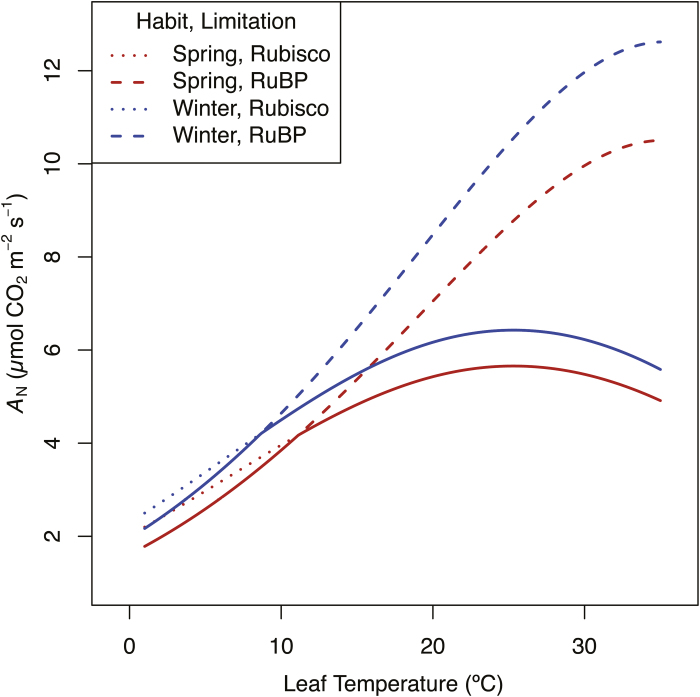
The photosynthetic temperature response for an average spring (lower curves) and winter (upper curves) ecotype. The dotted lines represent the Rubisco-limited photosynthetic rate, the dashed lines represent the RuBP-limited photosynthetic rate, and the solid lines represent the minimum of the two across the temperature range of 1–35 °C. The temperature response is modeled using the average *V*_Cmax_ and *J*_850_ measured at 25 °C for spring and winter habit ecotypes. The Rubisco-limited function was modeled according to a simple temperature response function (Equation 10; Bernacchi *et al.*, 2013) using a standard energy of activation constant for *V*_Cmax_ (Bernacchi *et al.*, 2001). The RuBP-limited function was modeled using a complete temperature response function (Equation 12, Bernacchi *et al.*, 2013), assuming an optimum temperature for *J*_max_ of 25 °C and utilizing the heat of activation, deactivation, and entropy constants of Bernacchi *et al.* (2003). All other Rubisco parameters (*K*_C_, *K*_O_, and Γ*) and *R*_d_ were modeled using the simple temperature response function (Equation 10; Bernacchi *et al.*, 2013), and heat of activation constants were taken from Bernacchi *et al.* (2001).

The winter habit ecotypes tend toward colder, more northerly latitudes than spring types, though the small number of ecotypes, coupled with imprecise geographic locations (Table 1), prevent accurate correlation of trait values and climate or latitude of origin. However, trait–latitude relationships suggested here were observed in *Populus balsamifera* where greater assimilation rates, electron transport, and mesophyll conductance were observed in genotypes from more northern latitudes, and a positive correlation was found between latitude and WUE (Soolanayakanahally *et al.*, 2009). Interestingly, LMA also acclimates in colder climates. Greater *A*_N_ and LMA are reported for lowland *Plantago* species compared with an alpine congener when grown at low temperatures (Atkin *et al.*, 2006). LMA increased at colder temperatures, effectively enhancing photosynthetic capacity per unit leaf area (Atkin *et al.*, 2006), allowing for positive carbon balance despite the absolute decline in photosynthesis with decreasing temperatures. Finally, we illustrate that photosynthetic trait–latitude responses were similar in Arabidopsis to that reported in *Populus* (Soolanayakanahally *et al.*, 2009) by modeling the response of assimilation to temperature in winter and spring ecotypes (Fig. 7). Not surprisingly, winter ecotypes are predicted to have higher *A*_N_ than spring ecotypes at all temperatures when grown and measured under identical conditions (i.e. at 25 °C) because both *V*_Cmax_ and *J*_850_ are greater in winter ecotypes.

### Conclusion

*Arabidopsis thaliana* ecotypes provide a powerful system for dissecting the mechanistic and genetic determinants of complex traits. *In vivo* estimates of photorespiration indicate that the molecular co-ordination of the Calvin–Benson cycle with the photorespiratory pathway scales to the physiological level as a correlation between photorespiratory CO_2_ efflux and CO_2_ assimilation. The absence of genetic correlation between photosynthesis and photorespiration is perhaps surprising given the strong physiological links between these processes (Busch *et al.*, 2018). Strong genetic correlations between mesophyll conductance and electron transport supporting carboxylation, and between maximum carboxylation and electron transport capacities, point to shared inheritance for traits underlying variation in photosynthesis among ecotypes. Structural and physiological traits were differentiated by ecotype life history strategy, with winter annuals generally exhibiting greater structural robustness, physiological capacity, and WUE. Integrated WUE was positively correlated with assimilation rate and mesophyll conductance in winter, but not spring, ecotypes, largely resulting from a lack of variance for *g*_sH_ in winter ecotypes and demonstrating that if *g*_sH_ is held relatively constant, WUE is responsive to mesophyll conductance.

## Supplementary Data

Supplementary data are available at *JXB* online.

Table S1. Mean (± SE) ecotype trait values

Fig. S1. A comparison of photorespiration estimated by two methods.

Fig. S2. Leaf thickness (*T*_L_) and leaf dry matter content (LDMC) of spring and winter annual ecotypes.

Supplementary Figures S1-S2 and Table I
