# Supplementary material for: Photorespiration differs among Arabidopsis thaliana ecotypes and is correlated with photosynthesis
Source: J Exp Bot. 2018 Jul 25;69(21):5191–204. doi: 10.1093/jxb/ery274 (PMC6184796; doi:10.1093/jxb/ery274)

Supplementary Table I. Mean (± se) ecotype trait values. Abbreviations and units are as indicated in Table II.

Ecotype	R_d	g_{sc}	g_m	g_{tot}	J_T	J_C	J_O
**Ag-0**	0.474 (0.0856)	0.146 (0.012)	0.701 (0.0659)	0.0463 (0.00384)	97.2 (7.49)	65.5 (4.92)	31.6 (2.8)
**Bil-5**	0.510 (0.0906)	0.123 (0.00893)	0.88 (0.0424)	0.0507 (0.00275)	125 (2.16)	81 (2.07)	44.5 (0.723)
**Bur-0**	0.224 (0.0574)	0.16 (0.00849)	0.769 (0.0494)	0.0511 (0.00188)	107 (3.79)	71.1 (2.27)	35.8 (1.9)
**Eden-1**	1.00 (0.154)	0.114 (0.00596)	0.746 (0.0569)	0.0443 (0.0029)	125 (3.52)	79.5 (2.34)	45.1 (2.22)
**Kas-1**	0.387 (0.0950)	0.138 (0.0124)	0.889 (0.0802)	0.0535 (0.00451)	125 (6.92)	81.5 (4.89)	43.6 (2.26)
**Knox-18**	0.338 (0.148)	0.132 (0.00759)	0.614 (0.0389)	0.041 (0.00196)	95.3 (4.63)	62.5 (2.8)	32.8 (1.89)
**Ler-1**	0.527 (0.110)	0.184 (0.0182)	0.831 (0.0153)	0.0559 (0.00174)	108 (3.32)	73.6 (1.92)	34 (1.6)
**NFA-10**	0.850 (0.117)	0.123 (0.0149)	0.593 (0.0565)	0.0396 (0.00399)	94.2 (9.73)	62.2 (5.9)	32 (4.04)
**Omo2-3**	0.521 (0.0584)	0.108 (0.0083)	0.688 (0.0551)	0.0415 (0.00308)	105 (2.44)	67.7 (2.28)	37.7 (0.843)
**Sq-8**	0.822 (0.139)	0.0983 (0.00754)	0.559 (0.0627)	0.0346 (0.00266)	109 (11)	67.2 (6.16)	42.1 (4.95)
**Tamm-2**	0.539 (0.184)	0.133 (0.00594)	0.743 (0.0313)	0.0465 (0.0012)	104 (4.06)	68.5 (2.39)	35.5 (1.77)
**Ts-1**	0.477 (0.190)	0.136 (0.0144)	0.695 (0.0599)	0.0453 (0.0042)	112 (10)	72.1 (6.28)	40.1 (3.84)
**Tsu-1**	0.188 (0.0546)	0.155 (0.0151)	0.752 (0.0559)	0.0498 (0.00409)	107 (8.45)	70 (5.32)	36.7 (3.27)
**Ws-2**	0.441 (0.134)	0.161 (0.0195)	0.705 (0.0599)	0.0483 (0.00421)	115 (6.87)	74.4 (4.53)	40.6 (3.08)

Supplementary Table I. Continued.

Ecotype	V_{Cmax}	J_{850}	A_N	PR	A_N/g_{sH}	δ^{13}C	N_{area}	LMA
**Ag-0**	51.3	96.5	11.9	3.96	51.9	-31.4	7.2	21.2
	(4.82)	(7.43)	(0.943)	(0.35)	(3.35)	(0.245)	(0.0726)	(1.26)
**Bil-5**	58.8	124	14.2	5.56	73.3	-28.6	6.54	23.2
	(2.79)	(6.67)	(0.529)	(0.0904)	(2.99)	(0.223)	(0.0658)	(0.555)
**Bur-0**	50.1	84.4	13.1	4.47	53.4	-30.8	6.3	19.6
	(1.61)	(3.26)	(0.45)	(0.237)	(2.56)	(0.355)	(0.148)	(0.666)
**Eden-1**	55.5	105	13.2	5.64	72.9	-29.1	6.73	23.4
	(2.77)	(9.02)	(0.599)	(0.277)	(2.33)	(0.321)	(0.0859)	(0.472)
**Kas-1**	51.3	86.9	14.6	5.45	66.4	-29.6	6.86	20.3
	(4.67)	(6.15)	(0.992)	(0.282)	(4.79)	(0.22)	(0.0901)	(0.904)
**Knox-18**	39.6	73.1	11.2	4.09	53.6	-31.1	6.97	17.6
	(3.1)	(4.08)	(0.482)	(0.236)	(3.17)	(0.198)	(0.0781)	(0.692)
**Ler-1**	54.8	102	13.6	4.26	48.6	-31.7	6.71	19.8
	(1.53)	(5.05)	(0.346)	(0.2)	(5.18)	(0.161)	(0.0924)	(0.736)
**NFA-10**	42.4	74.5	10.7	4	54.5	-31	7.01	17.9
	(2.5)	(4.98)	(1.04)	(0.505)	(3.14)	(0.272)	(0.0451)	(0.694)
**Omo2-3**	49.8	95.3	11.7	4.71	68.4	-29.6	6.31	19
	(1.65)	(4.65)	(0.578)	(0.105)	(2.79)	(0.235)	(0.0832)	(0.873)
**Sq-8**	41.8	80	10.7	5.26	62.3	-30.1	6.85	16.2
	(4.15)	(8.86)	(0.972)	(0.619)	(4.26)	(0.198)	(0.0885)	(0.6)
**Tamm-2**	50.9	103	12.2	4.44	57	-30.2	5.84	22.5
	(3.72)	(8.98)	(0.41)	(0.221)	(3.14)	(0.281)	(0.121)	(0.752)
**Ts-1**	45.6	84.5	12.5	5.01	58	-30.3	6.51	18.5
	(4.68)	(8.12)	(1.12)	(0.481)	(1.28)	(0.165)	(0.09)	(0.241)
**Tsu-1**	47.6	88.2	12.7	4.59	52.9	-31.6	6.93	15.5
	(3.74)	(7.04)	(0.963)	(0.409)	(4.23)	(0.202)	(0.1)	(0.503)
**Ws-2**	52.2	92.8	13.1	5.07	54.3	-31.4	7.01	17.1
	(5.08)	(8.74)	(0.946)	(0.386)	(3.17)	(0.181)	(0.159)	(0.401)

Supplementary Figure 1. The relationship between photorespiratory CO_2 release (PR) estimated with two differing methods. On the x-axis, PR was calculated with Equation-5 presented by Busch (2014) and is a combination of the instantaneous net assimilation rate (A_N), ecotype-mean respiration rate (R_d), the instantaneous [CO_2] in the chloroplast, and the CO_2 compensation point in the absence of R_d (Γ^*). The calculation of PR used throughout this manuscript is on the y-axis, and was calculated with a combination of the instantaneous calibrated electron transport rate, A_N, and R_d. A_N, C_c, and J_T were all measured at an ambient [CO_2] of 400 µmol mol^{-1}. Each ecotype is represented with a unique symbol. The dotted line represents unity. The solid line is a linear ordinary least-squares regression fit where the intercept (0.473) likely differs from zero (p = 0.06), the slope (1.0133) is significant (p < 0.001), and variance explained is high (R^2 = 0.796). Genetic variance in photorespiration as calculated on x-axis is roughly equal to that on the y-axis, but broad-sense heritability of PR is higher (H^2 = 0.321).

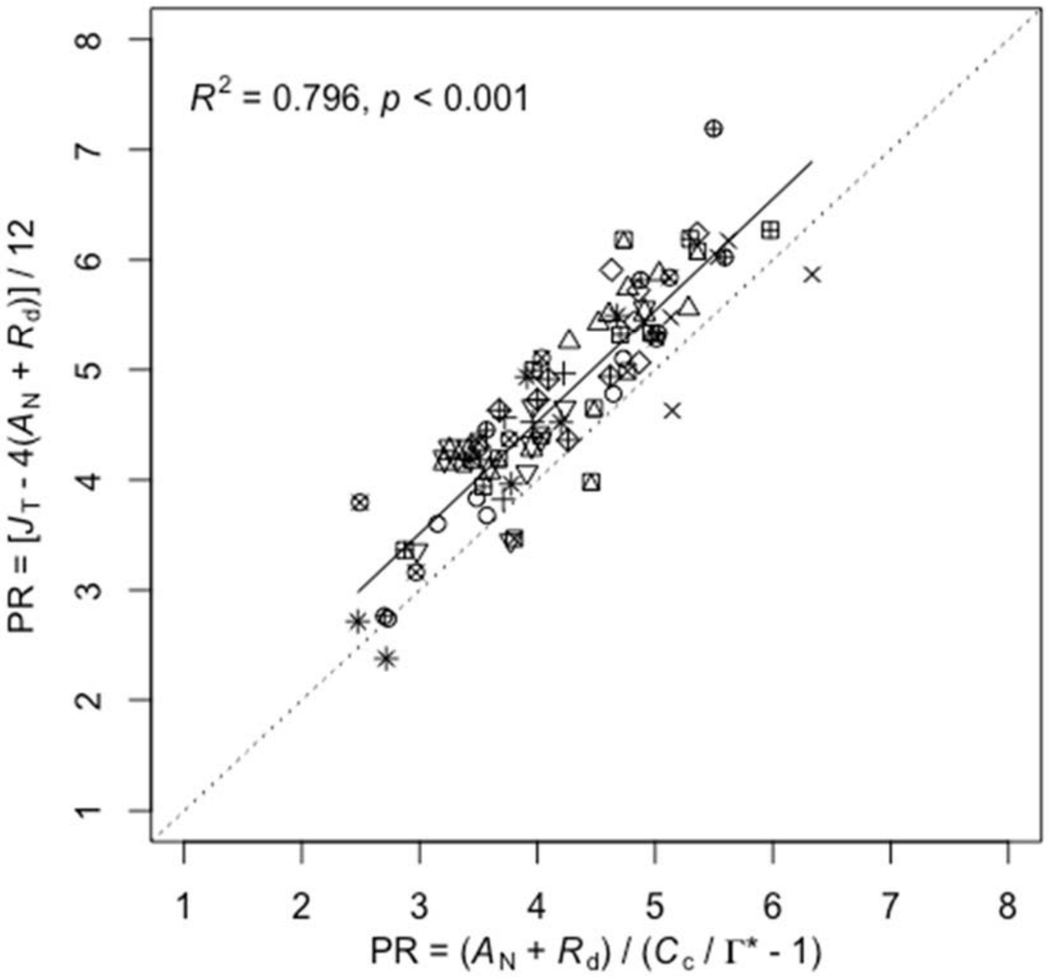

Supplementary Figure 2. Comparisons of trait values in ecotypes exhibiting spring or winter annual life-history strategies. The traits are (A) leaf thickness (T_L) and (B) leaf dry matter content (LDMC). Leaves were thicker and had greater LDMC in winter (n=6) relative to spring (n=8) ecotypes (*p*<0.001) following unequal-variance *t*-tests. Eight of the 14 ecotypes exhibit the spring habit and box width is scaled to the relative sample size in each group (*N*=46 for spring, *N*=34 for winter).

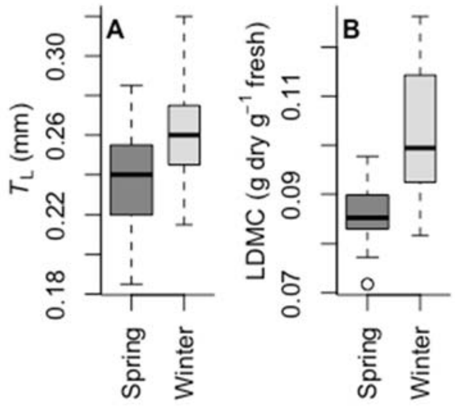

Supplement: Supplementary Figures S1-S2 and Table I [file ery274_suppl_supplementary_figures_s1-s2-and-table_i.pdf]
